# 3D absorbed dose distribution estimated by Monte Carlo simulation in radionuclide therapy with a monoclonal antibody targeting synovial sarcoma

**DOI:** 10.1186/s40658-016-0172-1

**Published:** 2017-01-18

**Authors:** David Sarrut, Jean-Noël Badel, Adrien Halty, Gwenaelle Garin, David Perol, Philippe Cassier, Jean-Yves Blay, David Kryza, Anne-Laure Giraudet

**Affiliations:** 1 Univ Lyon, INSA-Lyon, Université Lyon 1, CNRS, Inserm, CREATIS UMR 5220, U1206, Lyon, F-69008 France; 2 Univ Lyon, Centre Léon Bérard, Lyon, 69008 France; 3 Univ Lyon, Université Lyon 1, CNRS, LAGEP UMR 5007, Lyon, F-69008 France; 4 Hospices Civils de Lyon, Imthernat plateform, Lyon, F-69008 France

**Keywords:** Targeted radionuclide therapy, Absorbed dose estimation, Monoclonal antibody, Synovial sarcoma, Monte Carlo simulation

## Abstract

**Backround:**

Radiolabeled OTSA101, a monoclonal antibody targeting synovial sarcoma (SS) developed by OncoTherapy Science, was used to treat relapsing SS metastases following a theranostic procedure: in case of significant ^111^In-OTSA101 tumor uptake and favorable biodistribution, patient was randomly treated with 370/1110 MBq ^90^Y-OTSA101. Monte Carlo-based 3D dosimetry integrating time-activity curves in VOI was performed on ^111^In-OTSA101 repeated SPECT/CT. Estimated absorbed doses (AD) in normal tissues were compared to biological side effects and to the admitted maximal tolerated absorbed dose (MTD) in normal organs. Results in the tumors were also compared to disease evolution.

**Results:**

Biodistribution and tracer quantification were analyzed on repeated SPECT/CT acquisitions performed after injection of ^111^In-OTSA101 in 19/20 included patients. SPECT images were warped to a common coordinates system with deformable registration. Volumes of interest (VOI) for various lesions and normal tissues were drawn on the first CT acquisition and reported to all the SPECT images. Tracer quantification and residence time of ^111^In-OTSA101 in VOI were used to evaluate the estimated absorbed doses per MBq of ^90^Y-OTSA101 by means of Monte Carlo simulations (GATE). A visual scale analysis was applied to assess tumor uptake (grades 0 to 4) and results were compared to the automated quantification. Results were then compared to biological side effects reported in the selected patients treated with ^90^Y-OTSA101 but also to disease response to treatment.

After screening, 8/20 patients were treated with 370 or 1110 MBq ^90^Y-OTSA101. All demonstrated medullary toxicity, only one presented with transient grade 3 liver toxicity due to disease progression, and two patients presented with transient grade 1 renal toxicity. Median absorbed doses were the highest in the liver (median, 0.64 cGy/MBq; [0.27 −1.07]) being far lower than the 20 Gy liver MTD, and the lowest in bone marrow (median, 0.09 cGy/MBq; [0.02 −0.18]) being closer to the 2 Gy bone marrow MTD. Most of the patients demonstrated progressive disease on RECIST criteria during patient follow-up. ^111^In-OTSA101 tumors tracer uptake visually appeared highly heterogeneous in inter- and intra-patient analyses, independently of tumor sizes, with variable kinetics. The majority of visual grades corresponded to the automated computed ones. Estimated absorbed doses in the 95 supra-centimetric selected lesions ranged from 0.01 to 0.71 cGy per injected MBq (median, 0.22 cGy/MBq). The maximal tumor AD obtained was 11.5 Gy.

**Conclusions:**

3D dosimetry results can explain the observed toxicity and tumors response. Despite an intense visual ^111^In-OTSA101 liver uptake, liver toxicity was not the dose limiting factor conversely to bone marrow toxicity. Even though tumors ^111^In-OTSA101 avidity was visually obvious for treated patients, the low estimated tumors AD obtained by 3D dosimetry explain the lack of tumor response.

## Background

Synovial sarcomas (SS) are rare tumors accounting for 2.5 to 10% of all soft tissue sarcomas worldwide and for 2% of all malignant neoplasms, affecting mostly teenagers and young adults. Treatment rely on surgery and radiotherapy at initial stage, and chemotherapy (doxorubicin and/or ifosfamide) at metastatic stage with then a median survival of only 12 months.

Genome-wide gene expression profile analysis has revealed that the gene encoding frizzled homolog 10 (FZD10), a 7-transmenbrane receptor and member of the Wnt signaling receptor family, was overexpressed in SS, yet undetectable in normal human tissues excepting placenta [1–4]. OncoTherapy Science Inc. has developed a chimeric humanized monoclonal antibody (mAb) against FZD10, named OTSA101. In mouse xenograft model, DTPA ^90^Y radiolabeled OTSA101 (^90^Y-OTSA101) was shown to exhibit significant antitumor activity following a single intravenous injection [1] without significant toxicities, allowing for a first-in-man phase I trial.

This trial, named Synfrizz, was conducted on a theranostic model of radionuclide therapy with a two-phase approach: a screening phase followed by a therapeutic phase in case patient fulfilled the defined criteria. The screening phase evaluated clinical and biological parameters and studied the biodistribution and tumor avidity of ^111^In-OTSA101 on repeated SPECT/CT acquisitions. As usually observed in radioimmunotherapy, the liver concentrated a large amount of radioactivity and was at that time considered to be the organ at risk. Therefore, the treatment therapeutic window was evaluated with two parameters helping to screen the patients for therapy: tumor ^111^In-OTSA101 uptake intensity visually compared to mediastinal blood pool uptake on SPECT/CT acquisitions, and a liver estimated absorbed dose (AD) performed on repeated 2D whole body acquisitions. If at least one lesion demonstrated a tracer uptake greater than mediastinum and estimated liver AD would be less than liver MTD (20 Gy) when using the maximum activity of ^90^Y-OTSA101, patient was randomly treated with 370/1110 MBq ^90^Y-OTSA101 in the treatment phase.

Radionuclide therapy efficacy theoretically relies on a selective high and prolonged tumor uptake and a low normal tissue uptake with rapid wash-out of the vectorised therapeutic particle emitter radionuclide. This would result in high tumor AD and low normal tissue AD, widening the therapeutic window. This can only be evaluated on repeated scintigraphies over a long period of time, at least close to therapeutic radionuclide physical half-life. Radioactivity quantification in regions of interest drawn on tumors and normal tissues can be performed on planar scintigraphy (2D) but organs superposition limits its capacity to precisely approach AD. 3D quantification performed on SPECT/CT images has been proposed with improved results when compared to 2D, e.g., [5, 6] (among others), but is not yet widely available. Indeed, ADs may be estimated by means of several methods, such as the MIRD approach using S-values for doses at organ-level [7, 8], dose point kernel (DPK)-based convolution [9–11], or Monte Carlo (MC) for doses at pixel-level. MC is considered the reference method, and several comparisons with the others methods were performed and analyzed [10, 12–14].

In this paper, we present an ancillary retrospectively study focused on predicting the AD that would have been delivered by ^90^Y-OTSA101 based on 3D Monte Carlo AD estimation applied on diagnostic imaging performed in the screening phase of the trial, and compared our results to observed toxicity and disease evolution in treated patients.

## Methods

### Patients

From 2012 to 2014, 20 metastatic SS patients that could not be treated with any other treatment were enrolled in the phase I clinical trial, which was previously approved by local authorities (ANSM; ClinicalTrials.gov Identifier: NCT01469975). Ten patients fulfilled the criteria for radionuclide therapy. Two died before they could receive the treatment, leading to a total of eight treated patients. SPECT/CT data were gathered from 19 patients, with one patient excluded due to incomplete data. Patients’ characteristics will be described in a separate clinical publication reporting all the data obtained in the trial, as well as radiopharmaceuticals.

### Radiopharmaceutical

OTSA101-DTPA was labeled with ^111^In or ^90^Y according to a modified protocol [1]. Overall, 275 MBq of high purity ^111^In-chloride (specific activity >185 GBq/ *μ*g indium) in diluted hydrochloric acid (Covidien, Petten, The Netherlands) or 1665 MBq of ^90^Y-chloride (IBA-Cis bio, Saclay, France) were added to 2.25 mg of OTSA101-DTPA in the presence of acetate buffer and was incubated 90 min at 37 °C. At the end of the labeling, 0.8 mg of EDTA-2Na was added to the mixture solution. The radiochemical purity (RCP) was assayed with a gamma isotope TLC analyzer (Raytest, Courbevoie, France) using ITLC-SG (Biodex Tec-control black, Biodex, NY, USA) and 0.9% sodium chloride solution as mobile phase. ^111^In-OTSA101 or ^90^Y-OTSA101 remained at the origin, whereas unbound ^111^In or ^90^Y migrated with an Rf of 0.9–1. The radiochemical purity of radiolabeled OTSA101-DTPA was routinely over 90% before injection. In order to verify immunogenicity of humanized monoclonal antibody against FZD10, all patients were systematically followed up by evaluation of human anti-mouse antibodies. No immunogenicity has been observed.

### Imaging

The acquisition protocol comprised six SPECT/CT and whole body planar emission scans, acquired at time points 1, 5, 24, 48, 72, and 144 h following intravenous injection of approximately 185 MBq of ^111^In-OTSA101. The exact times of the six acquisitions were extracted from the image DICOM header. The first six patients’ images were acquired with a Philips BrightView XCT device, and the remaining images using a Tandem Discovery NM/CT 670 from GE Medical Systems with two heads. Indium-111 principal gamma ray emissions are at 171 and 245 keV. A double energy window scatter subtraction method was applied. The photopeaks in keV were in the range of 153.9–188.1 and 220.5–269.5, respectively, and the scatter window was 198.6–219.6. A medium energy general purpose/parallel (MEGP/PARA) collimator with hexagonal holes was employed. The acquisitions were performed with two table steps for a total of 30 min. For each step, a 180° step-and-shoot rotation was carried out, with 6° angle increment, providing 60 projection frames thanks to the two heads. Planar images were 1024 × 256 with scan velocity of 10 cm min ^−1^. The 45-s CT acquisitions were performed right after the SPECT acquisitions, covering a large part of the body (92 cm, from patient’s neck to below the pelvic region). They were reconstructed with 0.976562×0.976562×1.25 mm^3^ voxel size. More details regarding these devices are to be found in [15]. SPECT sampling was 4.18×4.18×4.18 mm^3^. SPECT images were reconstructed with the ordered-subset expectation maximization (OSEM) algorithm provided by the manufacturer. All images were reconstructed with the same software version (Xeleris 3.0) and parameter sets, with ten iterations and five subsets used. Attenuation correction was applied with attenuation maps derived from the CT images. Images were corrected using the “Resolution Recovery” package, while taking collimator-detector response functions into account.

### Image registration

To compensate for patient motion between acquisitions, deformable image registration (DIR) was performed between the time series’ first CT image, acquired at H0+1hour, and the five others. The DIR algorithm was based on B-splines with mutual information [16]. This method was previously reported in the literature, for example in [17, 18]. This step’s uncertainty was estimated at less than 2 mm. The five CTs were warped using the obtained deformation vector field (DVF). The six images were averaged in a single 3D image, denoted avCT enabling us to reduce noise. This last step being optional but has been found to provide superior image quality than initial CT. SPECT images were also warped with the same resampled DVFs in order to obtain motion compensated SPECT series [18]. Impact of breathing motion during images acquisition has not been evaluated here but is a source of additional uncertainty.

### Volumes of interest: organs and lesions

**Fig. 1:**
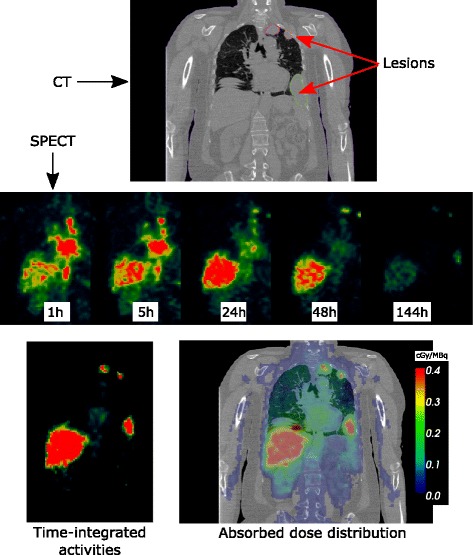
Illustration of initial data (CT and SPECT images), time-integrated activity distribution, and absorbed dose distribution

The analysis was focused on several volumes of interest (VOI): liver, spleen, heart, and bone marrow (BM), as well as the right and left kidneys. Contours were delineated on the first CT image. For BM, *L*_2_−*L*_4_ lumbar vertebrae were contoured as proposed in [19]. Among the studied patients, SS was generally associated with metastases comprising a large number of potentially identifiable lesions, mostly in the lungs. An expert physician (ALG) delineated a representative set of lesions (up to 25), regardless of the amount of activities depicted on SPECT images. Some lesions were selected owing to their high uptake values on SPECT, whereas others were chosen based only on avCT. Only lesions with a maximum diameter >1 cm were considered. For each patient, between 1 and 25 lesions were considered, resulting in a total of 95. For each VOI, volumes and mass were computed using the avCT images. Mass was estimated by converting Hounsfield units (HU) into mass, while taking into account the voxel volume of 1.19 mm^3^. Images are illustrated in Fig. 1.

### Analysis of the 3D activity distribution

We denote *A*_*x*_(*t*) the activity measured in voxel *x* at time *t*. Voxels were expressed in number of counts on the SPECT images. The conversion into activity (MBq) requires a calibration factor estimated by imaging a known activity amount, preferably under scatter and attenuation conditions close to patient imaging [8]. A patient-specific calibration factor (cps/MBq) was estimated by taking the total number of counts on the 1h SPECT image divided by the patient total activity weighted by the fraction of activity in the FOV (FAF). The FAF, which corresponds to the percentage of activity within the limited SPECT FOV, was estimated on the 1h whole body planar images. The SPECT FOV dimensions and localization were projected onto the planar images. Using the whole body images, the FAF was then calculated as the number of counts in this FOV divided by the total number of counts. The patient total activity at 1H was estimated by the injected activity, decay corrected, as the 1h images were acquired before urination. Activity was subsequently expressed in percentage of injected activity per kilogram of tissue (%IA/kg). For a VOI *h*, the total activity in the volume at instant *t* was obtained by summing up activities for all voxels belonging to *h*: $A_{h}(t) = \sum _{x} A_{x}(t) \; \forall x\in h$. These mean activities were associated with their standard deviation (SD) computed as $SD_{h}(t) = \sqrt {\sum _{x} A_{x}(t)^{2} - A_{h}(t)^{2}} \; \forall x\in h$. The SD corresponds to the activity heterogeneity within the VOI.

For the lesions, peak values were considered, because lesions were usually small (a few centimeters in diameter) and depicted partial volume effect tending to artificially reduce the activity near lesion boundaries. In analogy to the SUV-peak value for PET images [20], we defined $A_{h}^{\text {peak}}(t)$, the peak activity in VOI *h* at time *t*, as the mean activity measured in the spherical subregion contained in *h* exhibiting the maximum activity. The volume of this spherical region was set at 1 cc. In order to identify this peak subregion, SPECT images were convolved by a spherical mean filter kernel with a radius corresponding to the desired volume (in this case, about 6.2 mm to obtained a sphere of 1cc). The positions of the maximum voxel values in the time sequence of filtered images were averaged and the average position was used as the center of the peak sub-region. The value $A_{h}^{\text {peak}}(t)$ was defined as the mean activity in that subregion and expressed in %IA/kg. This method implicitly assumes that peak uptake locations in region are stable as a function of time. It was mostly verified for the data presented here, the standard deviation of the peak locations being low, around 5 mm. Taking into account the SPECT image resolution, the peak value is considered as stable.

Time-integrated cumulated activities in voxels *x* [7, 8, 21] were computed as follows: $\tilde {A}(x) = \int _{0}^{\infty } A_{x}(t) dt$. Like in [12], the integrals were approximated using with a two-step method. First, the trapezoid method was used on the first part of the curve, with the activity at injection time extrapolated with a linear fit towards 0 (uptake part). Second, the integration’s final part, from the last time point (H0+144 h) to infinity, was modeled using a fit of a mono-exponential function *A*_*x*_(*t*)=*A*_0_ *e* ^−*λt*^ of the curve’s last two or three points. Three points were generally used, except if the maximum uptake value was reached after the last three points. If activity increases in the last time point, an artificial time point is added to force the activity to decrease (at 60 h, with half of the maximum activity). Time-integrated activity $\tilde {A}_{h}$ for a VOI *h* was obtained with the same method applied to the mean *A*_*h*_(*t*) or peak activity $A_{h}^{\text {peak}}(t)$ and expressed in MBq h per mass and per MBq of injected activity: MBq h/kg/IA. The fitting procedure was performed with the weighted Levenberg-Marquardt optimization method and 100 iterations, with the weights being the standard deviation of the activities inside the VOI. Ceres-Solver [22] was used.

### 3D absorbed dose estimation

Absorbed dose distributions with ^90^Y were computed by Monte Carlo simulation using GATE [23, 24]. Time-integrated activity (TIA), i.e., the estimated total number of disintegrations, were estimated for all voxels and used as a 3D source map. ^111^In TIA, distribution was substituted with ^90^Y half-life, assuming that the biological half-life is the same between the two radionuclides. As ^90^Y undergoes *β* ^−^ decay (to stable ^90^Zr), the source was simulated as an electron source with isotropic emission and a continuous energy spectra obtained from the ^90^Y decay (mean of 933.7 keV, maximum of 2280.1 keV) simulated with Geant4, using the ENSDF database (Evaluated Nuclear Data Center, Brookhaven National Laboratory). While ^90^Y is known to also produce some gamma radiation (511 keV, 2.186 MeV), this was passed over because this amounted to less than <0.01% of yield. All electromagnetic processes were taken into account (Photoelectric, Compton, and Rayleigh scattering, pair production, ionization, bremsstrahlung, positron annihilation, multiple scattering) owing to the *emstandard_opt1* physic list of Geant4. Production cuts were set at 5 mm as differences with AD distribution obtained with lower values were negligible. Hounsfield units of the CT images were converted into patient material properties using Schneider’s method [25]. CT images were resampled like the SPECT images to 4.18^3^ mm^3^ voxel size in order to reduce computation time. AD distribution was recorded with a DoseActor [24] of the same voxel size. The simulations involved approximately 5×10^8^ emitted electrons. Statistical uncertainties were <1.5% for all voxels having >25% of the maximum AD by the patient. One simulation took about 20 h of computation time on single core of a conventional computer (PC, Linux, Intel Xeon CPU E5-1660, 3.3 GHz). The total 19 simulations were performed under 2h30 with a cluster of 200 CPUs. At the end of the simulation, the obtained AD distributions were scaled to correspond to the total number of disintegrations computed from the time-integrated image. The AD was expressed in cGy per administered activity (cGy/MBq). Mean AD in a VOI was computed by averaging the deposited energy in all voxels belonging to the VOI, and dividing by the total mass of the VOI.

### Visual grading

In analogy with the investigation of endocrine tumors [26, 27], a visual scale analysis was proposed. This procedure applied an uptake scoring scale comparing tumor uptake intensity to mediastinal blood pool background on SPECT-CT. Grade 0: no tracer uptake by tumor; grade 1: tumor tracer uptake lower than the mediastinum; grade 2: equal to the mediastinum; grade 3: greater than the mediastinum; and grade 4: equal to the most intense normal tissue uptake. In the phase I trial, patients were scheduled to be referred for ^90^Y-OTSA101 treatment if at least one lesion demonstrated a tracer uptake higher than mediastinum at any time of SPECT acquisition. Visual analysis was then compared to automated quantification based on the same set of rules, though computed as based on the AD estimation in the liver and the mediastinum. The scale is presented in Table 1.

**Table 1:** Grading scale for lesions, with uptake compared to mediastinum and liver uptake

Grade	Lesion uptake ratio
Grade 0	Lesion uptake < 20% mediast. uptake
Grade I	Lesion uptake < 80% mediast. uptake
Grade II	Lesion uptake > 80% and < 120% mediast. uptake
Grade III	Lesion uptake > 120% mediast. and < 80% liver uptakes
Grade IV	Lesion uptake > 120% liver uptake

## Results

*Toxicity*. The whole list of toxicities will be detailed in a separate clinical article. We focus here on the biological toxicity concerning the liver, kidney, and bone marrow functions using OMS grades of toxicity, evaluated on blood tests at D7, D14, and D28. Bone marrow toxicity as presented in Table 2 is separated in grades L (leucopenia, lymphopenia), T (thrombocytopenia), and A (anemia). Whole patients demonstrated significant medullary toxicity unresolved for 4/8 patients during follow-up. Only one patient had a grade 3 alteration of liver enzymes not related to treatment but to disease progression unresolved before patient death, and two patients presented with a transient grade 1 increased creatinine.

**Table 2:** Toxicity

Patient	Injected activity (MBq)	Liver AD (Gy)	Liver toxicity grade	Bone marrow AD (Gy)	Toxicity grade L	Toxicity grade T	Toxicity grade A	Kidney AD (Gy)	Kidney toxicity grade
2	370	1.49	0	0.17	2	0	1	0.25	0
3	1110	3.39	0	0.27	3	1	1	0.76	1
3 bis	1110	3.39	0	0.27	3	1	1	0.76	0
8	1110	8.78	0	1.47	3	1	3	2.27	1
10	370	2.51	0	0.33	1	0	1	0.76	0
11	1110	4.79	0	1.38	3	0	0	2.12	0
14	370	2.97	3	0.54	2	3	3	0.98	0
15	1110	11.22	0	1.33	4	3	2	2.48	0
20	1110	8.52	0	1.81	2	3	1	2.62	0

Absorbed dose (AD) are indicated in Gy for the liver, bone marrow, and kidneys. Toxicity grades are indicated for the liver, leucopenia/lymphopenia (L), thrombocytopenia (T), anemia (A), and kidneys. Patient 3 has been treated with two injections

*Grading*. In Table 3, the grades of all lesions exceeding grade 0 have been listed. The majority of visual grades corresponded to computed grades, excepted for five lesions. For one lesion (P11), a large difference between visual grading (IV) and computed grading (II) was observed.

**Table 3:** Visual (column 3) and computed (column 4) grading for lesions with grade higher than 0

P.	L.	V grade	C grade	Com.
5	1	I	I	
	2	III	III	
8 ^a^	1	III	III	
	2	III	III	
	3	IV	IV	
	4	IV	III	Lower grade
10 ^a^	1	III	II	Lower grade
	2	II	II	
	3	II	II	
	4	I	I	
11 ^a^	1	IV	II	Lower grade
	2	I	I	
	3	II	I	Lower grade
	4	I	I	
	5	I	I	
15 ^a^	1	II	I	
17	1	II	II	
20 ^a^	1	III	III	
	2	III	II	Lower grade

^a^Patients that have been indeed treated

**Fig. 2:**
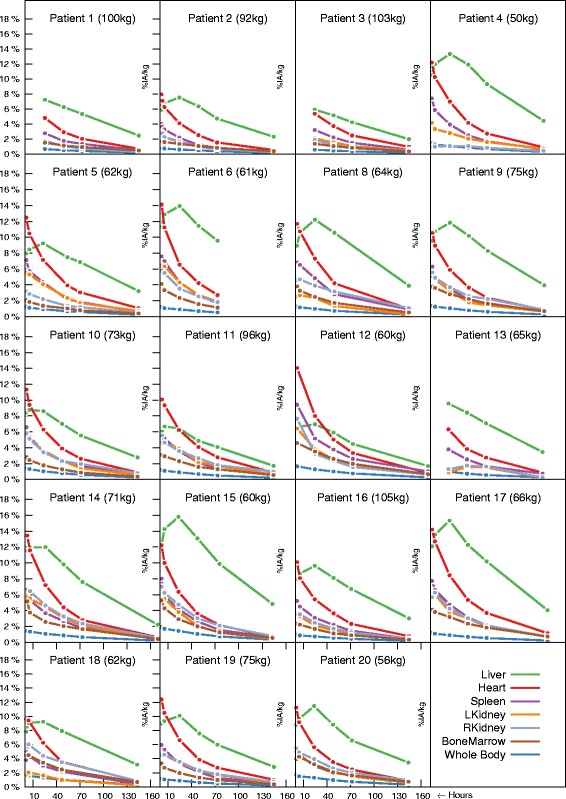
Variation of %IA/kg with time for several organs (liver, heart, kidneys, bone marrow, and spleen). The patient weight in kilograms is also displayed

*Biodistribution and kinetics*. ^111^In-OTSA101 biodistribution was similar to that usually observed in radioimmunotherapy with a predominant radiotracer uptake in the liver. Figure 2 shows the time activity curves of ^111^In-OTSA101 for different organs. Values are expressed in %IA/kg. Based on this figure, a similar behavior was observed with all VOI showing a monotonous decrease since the first time point, excepting the liver, which depicted a characteristic accumulation phase between 1 and 24 h after injection, followed by a clearance phase. Only three patients (P1, P3, P13) did not exhibit this accumulation phase, maybe situated between 5 and 24h. No particular uptakes by other organs than the liver were observed. Relative activities differed from patient to patient. Maximum values for liver uptake ranged from approximately 8% IA/kg (P3) up to >18% (P17). No clear correlation was found between the patient weights and accumulated activities (*r* = −0.75), yet there was a tendency towards smaller activities for higher patient weights. We also noticed the very fast clearance phase from the heart as commonly observed. Of note, the standard deviations of all activity points have not been displayed in this report, but ranged between 0.2 and 3.8%/kg, with the highest values observed for the heart.

**Fig. 3:**
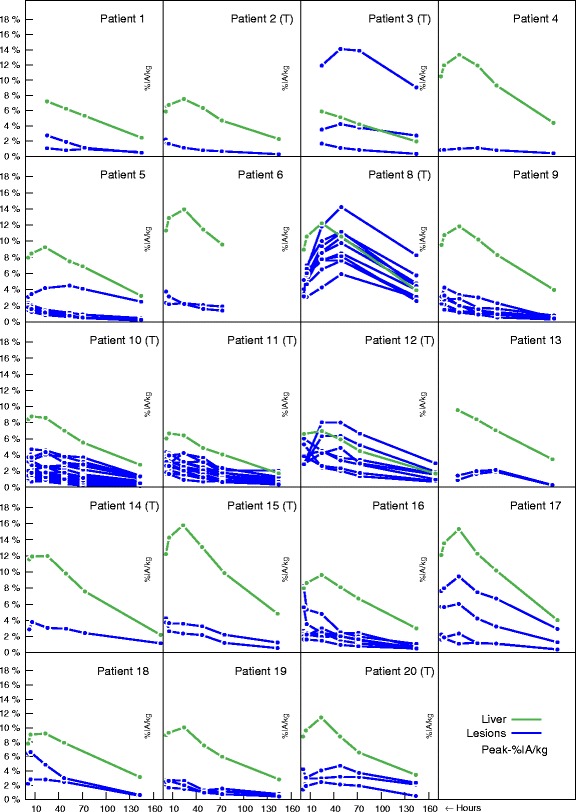
Variation of %peak-activity/kg with time for several lesions compared to the liver. Patients with a “T” indicated that they had been treated

Figure 3 displays the tracer kinetics as the % peak-activity/kg in relation with time for several lesions compared to the activity observed in the liver. Heterogeneous results were observed. Several lesions (P3, P5, P8, P12) exhibited typical two-phase curves with an initial accumulation phase reaching a maximum at around 24–48 h, thus at a later time point than the liver peak. By contrast, other lesions did not display an accumulation phase. Except for a few specific lesions, the activity concentrations in the lesions were usually lower than those in the liver. In contrast, several lesions in P3 and P8 showed very high uptake, suggesting an antibody accumulation in the tumor. Lesions smaller than 1 cm in diameter were not studied due to the limited resolution capacity of SPECT: their segmentation was found to be very difficult and unreliable.

**Fig. 4:**
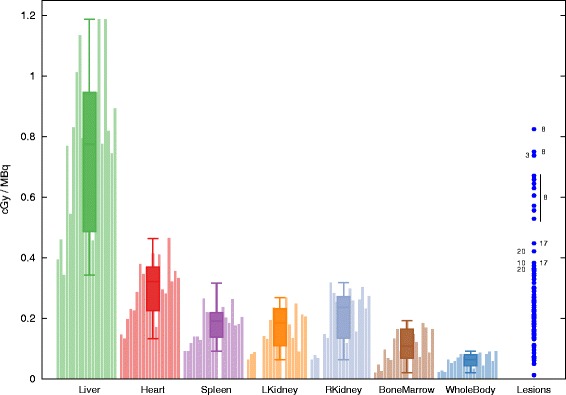
Estimated absorbed dose in cGy by injected activity (MBq) in several VOI and lesions for all patients. Patient number is indicated close to the few lesions with the largest absorbed doses

**Fig. 5:**
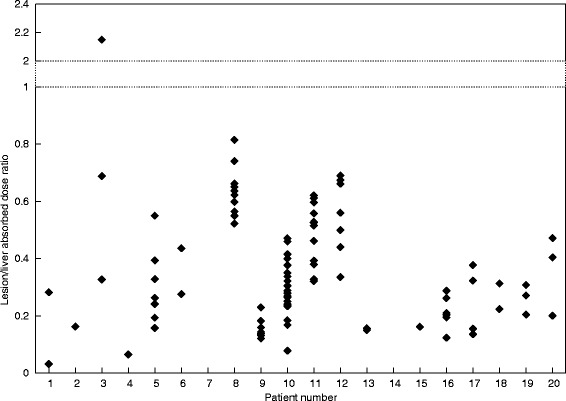
Ratio between absorbed doses by lesions and the liver for all patients

*Absorbed dose*. Median ADs were the highest in the liver (median, 0.64 cGy/MBq; [0.27–1.07]), followed by the mediastinal blood pool (median, 0.27 cGy/MBq, [0.13–0.41]), kidneys (median, 0.17 cGy/MBq, [0.05–0.27]), and bone marrow (median, 0.09 cGy/MBq, [0.02–0.18]). ADs to VOI are displayed in Fig. 4, and the ratios between lesions and liver in Fig. 5. The liver not only showed the highest values but also the largest standard deviation for all patients.

Lesions exhibits very heterogeneous values independently of the tumor size. The lesion uptakes in P3 and P8 were clearly visible. Figure 5 shows that no lesions, excepting one, displayed a higher AD than the liver. Large heterogeneity was observed, even within the lesions of the same patient. While several lesions demonstrated no radiotracer uptake and seemed not to capture the mAb, others showed good uptake (P8, P3). Estimated ADs in the 95 selected lesions ranged from 0.01 to 0.82 cGy per injected MBq (median, 0.25 cGy/MBq).

**Fig. 6:**
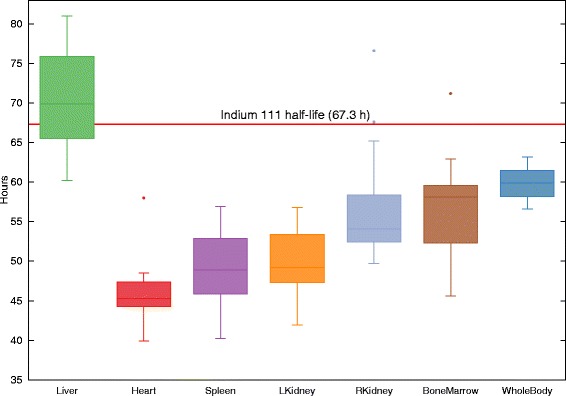
Effective half-lives of several organs (liver, heart, kidneys, bone marrow, spleen, and whole patient) for all patients. Physical half-life of ^111^Indium is indicated by the horizontal line

*Half-lives*. Figure 6 illustrates box plots of the effective half-lives for all organs and patients. The box depicts first and third quartiles, with the band inside the box representing the median, and the whiskers’ ends the minimum and maximum values. Outliers that were not included between the whiskers have been plotted as dots.

## Discussion

The main observation is that heterogeneous uptakes were observed among patients, with the liver presenting the most significant activity. In the following, we consider the ^90^Y-OTSA101 injection at 1110 MBq, namely the maximum injected activity, regardless if the patient was treated or not. Liver doses would range from 4.3 to 13 Gy. This intense radiophamaceutical uptake by the liver was expected as commonly observed in mAb radioimmunotherapy studies, e.g., [28]. However, those values are below the maximum tolerated dose (MTD), estimated around 20 Gy [29]. AD was generally low in other organs (in average 3.3 Gy to the heart, 2.1 Gy to spleen, 1.9 and 2.3 for the left and right kidneys), and below their respective MTD. The same threshold is usually applied to the kidneys and other tissues else than bone marrow and was never reached by the estimated doses. This correlates with the absence of significant liver or renal toxicity observed in the treated patients. However, for bone marrow, five patients would have received more than 1.8 Gy, which is close to the MTD, estimated around 2 Gy [30]. This threshold was obtained by Benua in 1962 who observed no unacceptable hematological complication in case of BMAD less than 2 Gy in ^131^I treated patients [31]. Patient to patient variation was large. We considered the inter-patient coefficient of variation (CV, standard deviation over mean): ADs for organs depicted CV between 33 and 42%, except for bone marrow where it was 50%. Considering that partial volume effect [32] was only partly corrected here (thanks to the resolution recovery option of the reconstruction software), caution should be taken about the AD to bone marrow that may be larger than the one computed here.

We performed PET and scintigraphic acquisitions using bremsstrahlung from ^90^Y to match true dose rate images in two patients. However, the too low activity in whole body and tumors stopped us to proceed to further acquisitions for the remaining treated patients. Any quantification would have been impossible as normal organs other than the liver as well as the lesions were not visible.

Considering only the eight patients that actually received ^90^Y-OTSA101 injection, large medullary toxicity were observed. However, unlike [33] who found that 3D BM dosimetry on SPECT incorporating radiobiological modeling data and applying Monte Carlo calculation tended to correlate with haematotoxicity, no correlation was clearly found between the AD and observed toxicities. There is suspicion of unchelated ^90^Y that could not be predicted by the ^111^In images, but it cannot be proven. Moreover, patients had a medical history of multiple pre-treatments that may affect myelotoxicity.

The observed ^111^In-OTSA101 uptakes were visually obvious for only few patients. Hence, we cannot confirm high FZD10 antigen expression for the majority of patients SS metastases. This is different to genes screening in SS lesions as FZD10 was found to be highly expressed in 8/13 SS tumors, expressed in 4/13 and absent in one [34]. For example, for patients demonstrating intense lesion uptake (P3, P8), AD was estimated to be approximately 8.5 and 11.5 Gy, respectively, when their livers would receive 3.8 and 11.1 Gy. Patient 3 disease was stabilized after the first injection allowing for a second injection 6 months later. Unfortunately, lesions had a 28% RECIST progression 2 months later and the patient died of profuse hemoptysis 3 months after the second injection. Except for these two patients, lesions of the remaining six treated patients received far less than 10 Gy which was expected to be insufficient especially in some patients with bulky tumors. By comparison, high tumors ADs has been recently evaluated as up to 468 Gy with radionuclide therapy using ^177^Lu-PSMA to treat prostate adenocarcinoma metastases, with great impact on disease-free survival [35]. Dosimetry results explain the poor clinical benefice observed. This would be related to a low FZD10 expression in SS metastases.

We observed a tendency towards a delayed ^111^In-OTSA101 uptake in the lesions, at a maximum of 24–48h. Only one lesion of the 19 patients showed a lesion-to-liver AD ratio >1 (P3). We observed in Fig. 6 that all organs excepting the liver showed effective half-life lower than the ^111^In physical half-life of 67.3 h, meaning that the mAb tended to be eliminated from these organs, as expected. For the liver, we observed an estimated effective half-life slightly exceeding the physical half-life for some patients. One assumption to explain this fact is an accumulation in that region due to the circulating mAb. Another assumption could be the degradation of the radiopharmaceutical. As expected, for the VOI “WholeBody” (when merging all the image voxels), the effective half-life was slightly lower than ^111^In half-life, most likely due to the natural physiological clearance. Considering lesions >0.5 cGy/MBq, there was one for P3 and seven for P8. Their effective half-lives was between 69 and 102 h, thus larger than the ^111^In half-life (data not shown). This observation may indicate a late mAb uptake and accumulation by these lesions.

Regarding the proposed tumor uptake grading, the majority of visual grades corresponded to the automated computed ones. However, two patients (P10, P11) that were treated, would not have been treated if the automated grading have been used. Indeed, the visual grade corresponded to the maximal tracer uptake observed at any time in the most ^111^In-OTSA101 avid lesion giving a sort of “SUVmax” when computed grades and therefore tumors AD result from the integrated time activity curve and reflect more the total tumor radioactivity exposition.

## Conclusions

In this study, the quantitative 3D analysis of the biodistribution and dosimetry of a radiolabeled monoclonal antibody against FZD10 was performed for the first time in SS metastic patients. A complete workflow including images calibration, deformable registration, time activity curves integration, and Monte Carlo simulation has been proposed and applied on 19 patients repeated SPECT/CT. The proposed method indirectly confirm FZD-10 antigen expression in some patients with SS metastases, but ^111^In-OTSA101 lesions uptake appeared too low in half of the patients on the basis of visual grading. On the basis of automated tumor uptake grading method two other patients would not have been treated as tumor/mediastinum ratio was finally too low. Estimated ADs in the liver and bone marrow explain biological toxicity, and the too low AD in the tumors explain the lack of tumor response. Patient-specific quantitative 3D biodistribution and dosimetry appears feasible and seems to be essential in the theranostic approach for predicting toxicity, defining activity prescription, and studying absorbed dose-effect relationships.
